# Facebook Addiction: Onset Predictors

**DOI:** 10.3390/jcm7060118

**Published:** 2018-05-23

**Authors:** Roberta Biolcati, Giacomo Mancini, Virginia Pupi, Valeria Mugheddu

**Affiliations:** Department of Education, University of Bologna, via Filippo Re 6, 40126 Bologna, Italy; r.biolcati@unibo.it (R.B.); virginia.pupi2@studio.unibo.it (V.P.); mughedduvaleria@gmail.com (V.M.)

**Keywords:** Facebook addiction, onset predictors, personality traits, social loneliness, emotional loneliness, life satisfaction

## Abstract

Worldwide, Facebook is becoming increasingly widespread as a communication platform. Young people especially use this social networking site daily to maintain and establish relationships. Despite the Facebook expansion in the last few years and the widespread acceptance of this social network, research into Facebook Addiction (FA) is still in its infancy. Hence, the potential predictors of Facebook overuse represent an important matter for investigation. This study aimed to deepen the understanding of the relationship between personality traits, social and emotional loneliness, life satisfaction, and Facebook addiction. A total of 755 participants (80.3% female; *n* = 606) aged between 18 and 40 (mean = 25.17; SD = 4.18) completed the questionnaire packet including the Bergen Facebook Addiction Scale, the Big Five, the short version of Social and Emotional Loneliness Scale for Adults, and the Satisfaction with Life Scale. A regression analysis was used with personality traits, social, family, romantic loneliness, and life satisfaction as independent variables to explain variance in Facebook addiction. The findings showed that Conscientiousness, Extraversion, Neuroticism, and Loneliness (Social, Family, and Romantic) were strong significant predictors of FA. Age, Openness, Agreeableness, and Life Satisfaction, although FA-related variables, were not significant in predicting Facebook overuse. The risk profile of this peculiar behavioral addiction is also discussed.

## 1. Introduction

In 2016, Facebook (FB) was the most popular social networking site (SNS) with 1712 million active users [1]. Facebook has long established its supremacy in terms of active members, with membership numbers steadily increasing by 17–20% annually. Every minute on FB, 510,000 comments are posted, 293,000 statuses are updated, and 136,000 photos are uploaded [2]. The growing FB expansion and the widespread acceptance of this social network suggest that some unique factors are capable of gratifying the needs of a large number of Internet users [3]. An increasing number of research studies [4,5] have shown that the most popular reasons for using FB are “communication-related”, such as social relationship management and companionship-seeking.

Despite the many positive aspects associated with SNS use, such as establishing and maintaining relationships [6], some individuals seem to be driven by inner and outer forces to use SNSs excessively and compulsively [7], with detrimental effects on one’s own quality of life. A compulsive use of social networking sites can lead to a specific addiction, particularly when the site usage becomes excessive or motivated by purposes of mood alteration [3]. Facebook addiction (FA) is defined as a subtype of Internet addiction (IA) [8], which falls into the cyber relationship addiction category (over-involvement in online relationships) defined by Young [9,10], which is yet to be acknowledged by scholars and clinical practitioners [11]. Despite many researchers defending the evidence that the FB overuse can lead to addiction, the diagnoses of FA is still controversial [12,13] and the DSM-V [14] does not yet include FA as an addiction disorder. Nevertheless, Kuss and Griffiths [15] argued that it may be plausible to speak specifically of a “Facebook Addiction Disorder” because of its addiction criteria, such as neglect of personal life, mental preoccupation, escapism, mood modifying experiences, and tolerance and concealment of the addictive behavior that seem to be present in some people who use this social networking excessively. The main consequences of maladaptive FB usage are difficulties with time perception and time management capabilities, as well as with work, study habits, and friendship [16].

### 1.1. FA and Personality Traits

Many studies suggested that personal traits play an important role in addictive behaviors. Some of these examined the relationship between the Big Five personality traits and Internet abuse [17]. A recent meta-analysis [18] pointed out that all the Big Five personality traits are significantly associated with Internet addiction. In particular, Openness to new experiences, Conscientiousness, Extraversion, and Agreeableness are negatively associated with “heavy Internet users” and seem to form protective factors. On the other hand, Neuroticism, which is the opposite of emotional stability and refers to difficulty managing stress and anxiety, was positively associated with IA and considered a risk factor. Specifically referring to SNSs, addictive tendencies have been reported to be positively related to Extraversion and negatively related to Conscientiousness: extroverts may use social media excessively for their social capital enhancement, and conscientious people assign lower priority to activities such as Facebook to fulfill other responsibilities they have undertaken [19]. Moreover, findings indicated that with SNS addiction, addictive tendencies have been positively related to Neuroticism [3] and evidence shows that SNSs may be a method of seeking support for such people [20]. People with higher scores on Neuroticism spend more time with social connections in their SNS communication compared with face-to-face interaction [3,20].

### 1.2. FA and Social and Emotional Loneliness

Koc and Gulyagci [21] argued that although many studies suggest an association between personality and SNSs addiction, such research is still in its infancy and needs more evidence on the addictive usage of Facebook and its correlates. Other psychosocial variables must be considered when explaining FA: if Neuroticism seems related with social difficulties in face-to-face communication, loneliness and wellbeing are supposed to play a major role in FA. Lonely individuals have a higher preference for online interaction, as they perceive that online communication might be the ‘‘Prozac of social communication” [22], relatively less risky and easier than face-to-face communication because of greater anonymity [5,23]. When people become isolated in the real world, the need for contact remains, and with the help of social network sites such as FB, making contact to reduce loneliness is easier. This social skills model [24] suggests that individuals who are lonely develop a preference for online interactions and may exhibit problematic Internet use.

Although loneliness has been studied as a unidimensional construct [25], Weiss [26,27] first described the multidimensional nature of loneliness by proposing two distinct types: social isolation (social loneliness) and emotional isolation (family and romantic loneliness). The first one is based on affiliation; the second refers to the feeling of intimacy. Based on Weiss’ conceptualization, a previous study [28] showed that social loneliness emerged as a strong predictor of FA, whereas family loneliness resulted in indirect negative effects; there was no evidence about the relationship with romantic loneliness.

### 1.3. FA and Life Satisfaction

Facebook addiction was negatively related to life satisfaction [11,29,30]. Some controversial results regarding the association between FA and life satisfaction have been reported. In general, IA has a negative impact on life satisfaction, whereas life satisfaction affects IA [31]. According to Ellison et al. [32], the relationship between personal satisfaction and SNS could be explained by accounting for satisfaction as an antecedent: individuals with lower levels of life satisfaction could seek to participate in online social networks to increase their personal well-being.

From the aforementioned literature, we attempted to deepen the understanding of psychosocial predictors of Facebook addiction. In particular, we aimed to explore correlations between FA, personality traits, social and emotional loneliness, and life satisfaction within the Italian population. Next, we investigated the predictive effects of individuals’ personality, social, family, romantic loneliness, and life satisfaction on FA.

Our first hypothesis considers the five personality factors (Conscientiousness, Openness, Extraversion, Agreeableness, and Neuroticism) as predictors of FA. We expected that Extraversion would positively predict FA, so that more extroverted users are more at risk of developing FA than no-extroverted people. Secondly, we supposed that Conscientiousness would negatively predict FA, representing a protective factor. Thirdly, we hypothesized Neuroticism as a positive predictor of FA, as people who are higher in Neuroticism may use SNS compulsively to avoid emotional instability and they depend on the SNS for social interaction.

The second hypothesis considers loneliness and FA. Specifically, we supposed that the social and emotional loneliness result would be predictive of FA. Loneliness should directly influence preferences for online interaction, as lonely individuals feel that they can interact with others and express themselves better online than they do offline [33].

Finally, the third hypothesis considers low life satisfaction as a negative contributor of FA, namely individuals experiencing dissatisfaction with life would use FB excessively to alleviate their discomfort and obtain psychological benefits.

## 2. Materials and Methods

Participants were contacted online, using an Internet questionnaire built with Google Forms, a survey-generating tool [34]. The sample was recruited using several methods, including mailing lists, newsgroups, and social networking sites, and they were asked to answer some questions on Facebook use. The questionnaire was drafted in Italian. No personal identifying information was collected. No fee was offered. To check and prevent a person from re-entering the survey site, the subject’s IP address was monitored. Data were collected in 2017. The Ethics Commission of the institution where the authors work approved this survey, which was conducted in agreement with the ethical norms laid down by the Italian National Psychological Association.

A total of 755 participants (female = 80.3%; *n* = 606) aged between 18 and 40 (mean = 25.17, SD = 4.18) filled in the questionnaires. All the subjects were Italian citizens and they were mainly born in the south (63.8%), whereas 31.0% came from the north, and 5.2% from central Italian regions. Single people accounted for 644 (85.3%); whereas 111 subjects in a relationship (14.7%). One respondent had no qualifications (0.1%), 54 (7.2%) had a junior high certificate, 288 (38.1%) had a high school diploma, 286 (37.9%) had a bachelor’s degree, 121 (16%) had a master’s degree, and five people (0.7%) had a specialization or PhD.

### 2.1. Measures

The questionnaire included a first section regarding age, sex, marital status, education level, occupation, and region of provenance.

#### Facebook Use

The section consisted of closed, self-reported questions that investigated the frequency of access to Facebook, the daily time spent on the social network and the number of FB friends. In detail, the questions were: (1) “How frequently do you log into Facebook?” (many times per day; at least once a day; at least once a month; very infrequently); (2) “On average, in one day, how much time do you spend on Facebook?” (less than 15 min; from 15 min to ½ h; from ½ to 1 h daily; 1–2 h; 2–3 h; 3–4 h; over 6 h); (3) “How many friends do you have on Facebook?” (fewer than 100 people; from 100 to 300; from 301 to 500; from 501 to 1000; from 1001 to 1500; from 1501 to 2000; over 2000 people).

The Bergen Facebook Addiction Scale (BFAS) [35] is an 18-item questionnaire with a 5-point scale ranging from “very rarely” to “very often”. The scale is unidimensional and reflects the six core elements of addiction: salience, mood modification, tolerance, withdrawal, conflict, and relapse (three items for each dimension). Higher scores indicate greater Facebook addiction. Cronbach alpha was 0.91 in this sample.

Big Five Inventory (BFI-10) [36] is the short version of the Big Five Inventory (BFI-44) and consists of 10 items, with two items per scale. The scale uses a 5-point Likert scale from 1 (strongly disagree) to 5 (strongly agree). Many scientists recognize the BFI is a unifying structure for the description and assessment of personality dimensions. These are Conscientiousness, Openness, Extraversion, Agreeableness, and Neuroticism.

The short version of the Social and Emotional Loneliness Scale for Adults (SELSA-S) [37] consists of 15 items selected from the original SELSA subscales [38] and produces scores for three sub-factors of loneliness: Social, Family, and Romantic (each comprising five items). Items were rated on a 5-point Likert scale ranged from 1 (strongly disagree) to 5 (strongly agree). The shorter version of SELSA can be useful in clinical and research settings, where a short and multidimensional measure of loneliness affords greater efficiency. Second, a shorter version of the SELSA that is similar in length to the most commonly used loneliness measure, the Unidimensional revised University of California, Los Angeles, Loneliness Scale, Version 3 (UCLA-3) [25], may encourage investigators to use a multidimensional approach to measuring loneliness. This recommendation influenced the scholars’ decision to include it within the current study. In this sample, the Cronbach alpha was 0.86 for the Family loneliness, 0.50 for the Romantic loneliness, and 0.80 for the Social loneliness.

Satisfaction with Life Scale (SWLS) [39] consists of 5 items designed to assess an individual’s general sense of satisfaction with their life as a whole on a 7-point Likert scale ranging from 1 (strongly disagree) to 7 (strongly agree). The reliability of the scale (Cronbach’s α) was 0.89.

### 2.2. Analytical Procedures

First, the frequencies and means were computed for each variable. To describe the relationships between the dimensions investigated, Pearson’s correlations were performed. Differences between men and women for Facebook use were computed using Chi-square test. Finally, linear regression analysis was performed.

## 3. Results

### 3.1. Facebook Use

Concerning Facebook access frequency, most of the participants, 642 (85%), declared they accessed FB “many times a day”, whereas 101 (13.4%) “at least once a day”, 9 people (1.2%) “at least once a month”, and only 3 participants (0.4%) very infrequently. Women (86.6%) declared they connected “many times a day” compared with men (78.5%) [χ^2^ (3, *n* = 755) = 9.20, *p* = 0.027].

Most participants used Facebook from half an hour to one hour daily (25,6%), followed by one to two hours (23.7%), fifteen minutes to half an hour (19.5%), two to three hours (16.2%), less than 15 min (6.2%), three to four hours (5.7%), and over six hours (0.9%). Most participants had a friends list of 501–1000 people (31%), followed by 301–500 people (24.8%), 100–300 people (19.9%), 1001–1500 people (13.5%), over 2000 (4,9%), 1501–2000 people (3.6%), and only the 2.4% of participants declared they had a list of fewer than 100 friends. No gender differences were found regarding time spent on FB and friends number.

### 3.2. Facebook Addiction, Personality, Loneliness, and Life Satisfaction

The BFAS total score ranged between 18 and 74 (mean = 28.84; SD = 10.49), with higher scores indicating a greater level of addiction. The FA level was relatively low as demonstrated in Table 1, which shows the mean scores of the six dimensions (the core elements of addiction), all below the average of the scale.

The BFAS did not report a specific cut-off score for a categorization of FA. However, in line with the more conservative approach suggested by some authors [36], 3.3% (25) scoring three or above on all six dimensions were defined as addicted. Means and standard deviations for all the study variables are presented in Table 2.

### 3.3. Relationships among Personality Traits, Social and Emotional Loneliness, Facebook Addiction, and Life Satisfaction

Pearson correlation analyses showed that BFA was significantly correlated with all the variables except for Extraversion (Table 3).

### 3.4. Predictors of Facebook Addiction

We employed linear regression analysis to determine prominent predictors of FA. The dependent variable was the BFAS score, whereas the independent variables were Personality Traits, Family, Romantic, Social Loneliness, and Life Satisfaction. Age and gender have been included as the control.

The model suggested that 20% of the variance in FA could be explained by six significant predictors: Conscientiousness, Extraversion, Neuroticism, Family Loneliness, Romantic Loneliness, and Social Loneliness [*F*(10,754) = 18.20, *p* < 0.001]. All these variables positively predicted FA except for Conscientiousness that negatively predicted FA. Agreeableness, Openness and Life satisfaction as well as Age and Gender were not significant predictors (Table 4).

## 4. Discussion

The current study aimed to deepen the understanding of the relationship between Facebook addiction, personality traits, social and emotional loneliness, and life satisfaction within the Italian population. Specifically, the study investigated the predictive effects of individuals’ personality, social, family, romantic loneliness, and life satisfaction on FA. The findings showed that specific personality traits (Conscientiousness, Neuroticism, and Extraversion), and social and emotional loneliness (family, romantic, and social loneliness) could correctly predict the individuals’ likelihood of becoming Facebook addicted.

In general, FB was widely used by the participants. In our survey, the prevalence of FA observed was not alarming, but notably, we used the most conservative approach for the estimate. Specifically, the prevalence of Facebook addicted subjects found in the present study confirmed by previous works [4,40,41] showing that FA appears to range between 2 and 10% among adults.

As concerns onset predictors, findings revealed the following profile: individuals with low Conscientiousness, high Extraversion, and high Neuroticism who suffer from emotional and social loneliness are prone to becoming Facebook-addicted. In general, the results appear to support Caplan’s social skills model [42] of generalized problematic Internet use, according to which individuals probably use the medium to find social support or pain relief.

In particular, according to several studies [6,21], we provided evidence of the link between loneliness and FA. Lonely individuals turn to FB to find companionship [43] and relief from problems and worries related to socialization [44]. Indeed SNSs, such as FB, which is thought to be able to meet the psychological and emotional needs of the individual, have become crucial in many aspects of everyday life, such as socializing, alleviating loneliness, and establishing new relationships with the opposite sex [45]. Social compensation theory, which explains that people use social networking sites to meet their interpersonal needs that are unsatisfied in everyday life [46], provided an informative explanation, by means of an understanding of the link between FA and social and emotional loneliness. According to this perspective, individuals may use their online companionship to compensate for the lack of affective relationships in their actual social lives [34,47].

Moreover, in line with Andreassen et al. [48], the results confirmed that only some personality traits, namely Conscientiousness, Extraversion, and Neuroticism, are strong predictors of FA, suggesting that the associations between the behavioral addictions and personality traits vary depending on the different addictions considered. Regarding this, the results established that Conscientiousness was negatively related with FA in line with previous studies [36]. Conscientiousness seems to represent a protective factor for some so-called unproductive behavioral addictions, to distinguish them from so-called productive addictions, such as workaholism [48], FA, video gaming addiction, or general Internet addiction. Conscientiousness represents the tendency to be reliable, responsible, organized, and self-disciplined. Conscientious people were well self-controlled and tended to be careful publishing their statuses [49]; thus, they were not likely to be addicted to Facebook. The rationale is that if a highly conscientious person believes FB does not drive efficiency or production, they have decreased behavioral intentions towards it [50]. In other words, conscientious individuals will not invest a large amount of resources in FB because they prefer to stick to their main goals by avoiding distractions [51].

According to Hill et al. [52], Extraversion is more generally related to Internet addiction because extroverted individuals seem to seek stimulation and the media offer the ideal platform to seek stimuli [53]. Extraversion refers to the extent to which individuals are social, cheerful, active, and talkative. Individuals higher in Extraversion are expected to engage in high amounts of social interaction and approach others more easily [51]. FB simply provides another platform for extraverts to communicate with friends and contacts made off-line [54]. Notably, in our study Extraversion per se is not correlated with FA but is a predictor in the regression model when it is combined with other variables such as Neuroticism, low Conscientiousness, and the experience of loneliness. The lack of correlation leads us to interpret this result with caution.

Moreover, Neuroticism was a significant predictor of FA. Neuroticism is known to be a risk factor for the development of psychological illness in general [55]. Several kinds of behavioral addictions positively associated with Neuroticism can result from underlying features of social anxiety, low self-esteem, or fear of failure [56]. Indeed, social networking communication is preferable to daily offline communication for individuals with a high Neuroticism trait due to their social anxiety [57].

Our findings also revealed that FA is correlated with lower Life satisfaction [15]. Despite this, although some scholars considered low levels of well-being as a potential risk factor for problematic FB use [7], our results did not confirm the predictive effects of life satisfaction, showing that FA is rather explained by other aspects of psychological distress, such as social and emotional loneliness [47]. The SWLS produces an index of general satisfaction with life. Thus, it is plausible that life satisfaction is not a precursor of FA but rather a consequence of loneliness and neuroticism, and may play a mediating or moderating role.

Although correlated, neither age, Agreeableness, nor Openness were FA predictors. This means that the other personality variables and the three forms of loneliness have a stronger effect in explaining FA.

This research has some limitations that ought to be borne in mind for future research. First of all, the study is essentially correlational and causation cannot be inferred without hazard. That is, although the results suggest statistical predictive effects, they should be interpreted with caution and the hypothesis should be more properly tested in a longitudinal model. Moreover, in our study, the sample size was quite large and several correlations are rather small; thus, some of the significant results may have been driven by the large sample size. Our regression model only accounted for 20% of the variance in Facebook addiction. This means that other predispositions lead to FA, which future studies should consider.

Secondly, all the data in the present study were based on self-reporting, so the results may have been influenced by common method bias. Furthermore, one scale testing the romantic loneliness scale exhibited low internal consistency.

Finally, the convenience sampling and gender and age imbalance limit the generalizability of the findings. In the future, the same study should be replicated with a more representative sample.

## 5. Conclusions

Notwithstanding these limitations, the present study extends the previous FA literature showing that some personality traits and loneliness are dominant predictors of social media addiction.

We stress the importance of understanding the mechanisms behind FA for prevention and treatment. This line of research fits with the wider theoretical framework of the Person-Affect-Cognition-Execution (I-PACE) [58] model aimed at summarizing the mechanisms underlying the development specific Internet-use disorders in terms of a process model of the addiction. Indeed, we explicitly focused on predisposing factors, which could make individuals vulnerable to developing FA. According to I-PACE, future efforts will focus on the understanding of interactions between personal characteristics and cognitive and affective processes, namely other variables that could act as moderators and mediators within the addiction process, such as dysfunctional coping strategies, and FB expectancies. From a clinical perspective, those individuals addicted to using Facebook experience symptoms similar to those experienced by those who suffer from addiction disorders. Like other behavioral addictions, the aim of FA treatment cannot be total abstinence from using SNSs, since the latter is an integral element of today’s social culture. A suitable therapy goal is the controlled use of social networking applications, and understanding and working on the authentic interpersonal needs underlying compulsive overuse. In short, it is necessary now for clinicians to pay more attention to some of social media’s basic features that provide psychological gratification and compensation for individual interpersonal needs and their specific links with behavioral addiction.

## Figures and Tables

**Table 1:** Results of descriptive of the Facebook Addiction Scale dimensions.

Dimension	Min.	Max.	Mean	SD
Tolerance	1	5	1.84	0.80
Mood modification	1	5	1.78	0.91
Salience	1	4.67	1.58	0.67
Relapse	1	4.67	1.57	0.77
Conflict	1	4.67	1.51	0.66
Withdrawal	1	5	1.34	0.61
Mean of the BFAS total score	1	4.11	1.60	0.58

**Table 2:** Results of descriptive of the variables of interest.

Variable of Interest	Mean	SD
Conscientiousness (1, 5)	3.64	0.74
Openness (1, 5)	3.69	0.85
Extraversion (1, 5)	3.07	0.80
Agreeableness (1, 5)	4.00	0.64
Neuroticism (1, 5)	3.06	0.87
Family Loneliness (1, 5)	1.69	0.81
Romantic Loneliness (1, 5)	2.42	1.23
Social Loneliness (1, 5)	1.97	0.74
SWLS (5, 35)	23.76	6.34

**Table 3:** Pearson Correlation coefficients among all the variables.

Variable	1	2	3	4	5	6	7	8	9	10	11
1. Age (18, 40)	–										
2. Conscientiousness (1, 5)	0.149 **	–									
3. Openness (1, 5)	0.005	0.153 **	–								
4. Extraversion (1, 5)	−0.032	0.064	0.106 **	–							
5. Agreeableness (1, 5)	0.070	0.337 **	0.134 **	0.052	–						
6. Neuroticism (1, 5)	−0.089 *	−0.223 **	−0.133 **	−0.057	−0.280 **	–					
7. Family Loneliness (1, 5)	0.042	−0.138 **	−0.019	−0.074 *	−0.172 **	0.238 **	–				
8. Romantic Loneliness (1, 5)	−0.113 **	−0.152 **	0.024	−0.030	−0.144 **	0.164 **	0.192 **	–			
9. Social Loneliness (1, 5)	0.065	−0.153 **	−0.145 **	−0.237 **	−0.211 **	0.270 **	0.401 **	0.206 **	–		
10. SWLS (5, 35)	−0.083 *	0.241 **	0.139 **	0.194 **	0.181 **	−0.350 **	−0.408 **	−0.242 **	−0.382 *	–	
11. BFA (18, 74)	−0.091 *	−0.289 **	−0.098 **	0.024	−0.131 **	0.260 **	0.229 **	0.229 **	0.271 **	−0.242 **	–

Note: BFA = Bergen Facebook Addiction, SWLS = Satisfaction with Life Scale, * *p* < 0.05, ** *p* < 0.01.

**Table 4:** Results of linear regression analysis of Bergen Facebook Addiction (*n* = 755).

Dependent Variable	Independent Variable	β	*t*-Value	*p*
BFA	Age	−0.05	–1.56	0.118
	Gender	−0.03	−0.94	0.348
	Conscientiousness	−0.21	–5.71	0.000
	Openness	−0.03	−0.94	0.350
	Extraversion	0.10	2.91	0.004
	Agreeableness	0.05	1.31	0.190
	Neuroticism	0.12	3.33	0.001
	Family Loneliness	0.08	2.16	0.031
	Romantic Loneliness	0.12	3.38	0.001
	Social Loneliness	0.16	4.22	0.000
	SWLS	−0.04	−0.90	0.367

Note: β = Standardized coefficients.
